# *Thymus algeriensis* and *Thymus fontanesii*: Chemical Composition, In Vivo Antiinflammatory, Pain Killing and Antipyretic Activities: A Comprehensive Comparison

**DOI:** 10.3390/biom10040599

**Published:** 2020-04-13

**Authors:** Mansour Sobeh, Samar Rezq, Mohammed Cheurfa, Mohamed A.O. Abdelfattah, Rasha M.H. Rashied, Assem M. El-Shazly, Abdelaziz Yasri, Michael Wink, Mona F. Mahmoud

**Affiliations:** 1 AgroBioSciences Research Division, Mohammed VI Polytechnic University, Lot 660–Hay MoulayRachid, Ben-Guerir 43150, Morocco; aziz.yasri@um6p.ma; 2 Institute of Pharmacy and Molecular Biotechnology, Heidelberg University, Im Neuenheimer Feld 364, 69120 Heidelberg, Germany; 3 Department of Pharmacology and Toxicology, Faculty of Pharmacy, Zagazig University, Zagazig 44519, Egypt; samar_rezq@yahoo.com; 4 Departement of Biology, Faculty of Nature, Life and Earth Sciences, University of Djillali Bounaama, Khemis Miliana Road Teniet Elhad, Khemis Miliana 44225, Algeria; abou_yazid@hotmail.fr; 5 Laboratory of Natural Bioresources, Department of Biology, Faculty of Science, University of Hassiba Ben Bouali Chlef, Box 151, Chlef 02000, Algeria; 6 College of Engineering and Technology, American University of the Middle East, Kuwait; mohamed.abdelmoety@aum.edu.kw; 7 Department of Pharmaceutical Chemistry, Faculty of Pharmacy and Biotechnology, German University in Cairo, Cairo 11835, Egypt; rasha.rashied@gmail.com; 8 Department of Pharmacognosy, Faculty of Pharmacy, Zagazig University, Zagazig 44519, Egypt; assemels2002@yahoo.co.uk

**Keywords:** *Thymus algeriensis*, *Thymus fontanesii*, antioxidant activity, anti-inflammatory, antipyretic, analgesic, HPLC-PDA-ESI-MS/MS

## Abstract

This study aimed to investigate the chemical composition, and evaluate the antioxidant, anti-inflammatory, anti-pyretic, and the analgesic properties of methanol extracts from the leaves of *Thymus algeriensis and Thymus fontanesii* (Lamiaceae). Thirty-five secondary metabolites were characterized in both extracts using HPLC-PDA-ESI-MS/MS. Phenolic acids, mainly rosmarinic acid and its derivatives, dominated the *T. algeriensis* extract, while the phenolic diterpene carnosol and the methylated flavonoid salvigenin, prevailed in *T. fontanesii* extract. Molecular docking study was carried out to estimate the anti-inflammatory potential and the binding affinities of some individual secondary metabolites from both extracts to the main enzymes involved in the inflammation pathway. In vitro enzyme inhibitory assays and in vivo assays were used to investigate the antioxidant and anti-inflammatory activities of the extracts. Results revealed that both studied *Thymus* species exhibited antioxidant, anti-inflammatory, analgesic, and antipyretic effects. They showed to be a more potent antioxidant than ascorbic acid and more selective against cyclooxygenase (COX-2) than diclofenac and indomethacin. Relatively, the *T. fontanesii* extract was more potent as COX-2 inhibitor than *T. algeriensis*. In conclusion, *Thymus algeriensis* and *Thymus fontanesii* may be interesting candidates for the treatment of inflammation and oxidative stress-related disorders.

## 1. Introduction

Inflammation is a defense reaction of the body against the invasion of pathogens, injury, and other noxious stimuli. However, it can lead to persistent tissue damage by the migrated leukocytes or lymphocytes. Accordingly, it is essential to control inflammation and the associated pain and hyperthermia [1]. The most common treatments of inflammation currently utilize non-steroidal anti-inflammatory drugs (NSAIDs), central analgesics (opioids), glucocorticoids, and adjuvants drugs such as anticonvulsants and antidepressants. However, these agents have several side effects such as gastrointestinal disorders and are ineffective for treating some conditions. Further research for new bioactive molecules against pain and inflammation is, therefore, a persistent demand so that many studies are focusing currently on natural products as therapeutic alternatives [2].

The mint family Lamiaceae, comprises 236 genera, among them the genus *Thymus,* with 400 species of aromatic herbaceous, perennials, and small shrubs. They are cultivated originally in Europe but are now widely distributed in the Mediterranean region and Asia. Plants from the genus *Thymus* is used traditionally as herbal teas, culinary spices, condiments, and as analgesic, anti-rheumatic, antiseptic, astringent, and diuretic agents. Additionally, the plants are considered an important source of essential oils and the thyme oil, with its diverse biological properties including antioxidant, antibacterial, antimycotic, and age-delaying properties, is included in the world’s top ten essential oils [3].

The herbaceous fragrant, *Thymus algeriensis*, is an endemic plant to Libya, Tunisia, Algeria, and Morocco and considered as the most widespread North African species. The plant is largely used as a culinary herb and as a traditional medicine to treat digestive and respiratory infections (e.g., gastrointestinal dysentery, colds), prostate adenoma, and to prohibit abortion [3,4].

The spontaneous plant, *Thymus fontanesii*, is native to Algeria and Tunisia. Traditionally, it is used as a processed food preservative and as antispasmodic, carminative, stomachic, expectorant, antitussive, antiseptic, anthelmintic, and to treat some gastrointestinal diseases as well.

In the current study, the chemical composition of the methanol extracts from the leaves of *T. algeriensis* and *T. fontanesii* were characterized by LC-MS. Molecular docking in silico study was carried out to evaluate the anti-inflammatory potential and the binding affinities of some identified compounds in both extracts to the main enzymes involved in the inflammation pathway namely; cyclooxygenase-1 (COX-1), cyclooxygenase-2 (COX-2), 5-lipoxygenase (5-LOX), and 5-lipoxygenase activating protein (FLAP). The antioxidant, anti-inflammatory, anti-pyretic, and the analgesic properties of the extracts were evaluated both in vitro and in vivo.

## 2. Materials and Methods

### 2.1. Plant Material and Extraction

Leaves of *Thymus algeriensis* Boiss. et Reut. and *Thymus fontanesii* Boiss. et Reut. were collected in March 2018 from Algeria, “Ain Dem- AIN DEFLA” region (Latitude: 36.3059434N, Longitude: 2.5099262E, Altitude: 748 m) and “Mekhatria-AIN DEFLA” region (Latitude: 36.3025793N, Longitude: 1.9597495E, Altitude = 263 m), respectively. Voucher specimens were deposited in the herbarium of Pharmacognosy Department, Faculty of Pharmacy, Zagazig University, Egypt (Voucher specimen No. TA-L10, TF-L11).

Shade dried leaves (250 g) of each plant were ground and extracted by methanol 80% (3× 1L) at room temperature. The extracts were filtered, evaporated under vacuum and subjected to freeze-drying yielding 5 and 4.5 g of dried extract for *T. algeriensis* and *T. fontanesii,* respectively.

### 2.2. HPLC-PDA-ESI-MS/MS

A ThermoFinnigan LCQ-Duo ion trap mass spectrometer (ThermoElectron Corporation, Waltham, MA, USA) with an ESI source (ThermoQuest Corporation, Austin, TX, USA) was used to identify the phytochemical composition of the leaf extract as previously reported by Ghareeb et al. [5].

### 2.3. In Vitro Studies

#### 2.3.1. Cyclooxygenase (COX) Inhibition Assay

The potential of *T. algeriensis* and *T. fontanesii* extracts to inhibit ovine COX-1 and COX-2 was evaluated using an enzyme immunoassay (EIA) kit (Cayman Chemical, AnnArbor, MI, USA) according to the manufacturer’s instructions and the study by Adellall et al. [6].

#### 2.3.2. Lipoxygenase (LOX) Inhibition Assay

The potential of *T. algeriensis* and *T. fontanesii* extracts to inhibit lipoxygenase was evaluated using a lipoxygenase inhibitor screening assay kit (Cayman Chemical, AnnArbor, MI, USA) according to the manufacturer’s instructions and the study by Adellall et al. [6].

#### 2.3.3. Total Antioxidant Capacity (TAC) Assay

The total antioxidant capacity of the extracts was evaluated against ascorbic acid as a reference standard using the commercially available TAC ELISA kit (MBS726896, my BioSource, Inc., San Diego, CA, USA) according to the manufacturer’s instructions and according to Sobeh et al. [7].

### 2.4. Animals

Male albino mice (20–25 g) and Wistar rats (200–220 g) obtained from the faculty of Veterinary Medicine, Zagazig University, Zagazig, Egypt, were used in the experiments. The animals were settled in cages at constant standard environmental conditions and had free access to food and water *ad libitum*. All experimental protocols were approved by Zagazig Ethical Committee for animal use and care, Zagazig university, Zagazig, Egypt (Approval number: ZU-IACUC/3/F/115/2018).

#### 2.4.1. The Anti-Inflammatory Activity in Carrageenan-Induced Hind-Paw Edema Model

Nine groups of male rats (200–220 g) with five animals per group were used in this experiment. Induction of edema in the rats’ right hind paw was done by injecting 0.1 mL freshly prepared carrageenan solution (1% in 0.9% NaCl), into the sub-plantar tissue. An hour earlier, the rats received, orally, the vehicle (10 mL/kg), *T. algeriensis* and *T. fontanesii* extracts in three dose levels (200, 400, and 600 mg/kg), diclofenac (20 mg/kg), or dexamethasone (2 mg/kg). By the aid of a caliper ruler, the paw thickness (mm) was measured in the dorsal- plantar axis before and after injecting the carrageenan solution, and at hourly intervals for 5 h, and finally at 24 h. The cumulative anti-inflammatory effect during the whole observation period (0–24 h) was determined by calculating the area under the changes in paw thickness-time curve (AUC_0–24_).

#### 2.4.2. Leukocytes Recruitment into Peritoneal Cavity in Mice

Swiss albino mice (*n* = 5–8/group), were treated orally with the vehicle (1 mL/100 g), or *T. algeriensis* and *T. fontanesii* extracts in three dose levels (200, 400, and 600 mg/kg), 30 min before the intraperitoneal injection of 0.1 mL carrageenan solution (500 µg/mice) or 0.1 mL sterile saline. Diclofenac (p.o., 20 mg/kg) and dexamethasone (p.o., 2 mg/kg) were used as reference standards. Three hours later, the mice were euthanized, and each peritoneal cavity was washed with 3 mL of phosphate-buffered saline (PBS) containing 1mM ethylenediaminetetraacetic acid (EDTA). The total leukocytes count was determined in each peritoneal cavity wash using a hemocytometer and presented as number of cells/mL.

#### 2.4.3. Acetic Acid-Induced Vascular Permeability

Acetic acid-induced vascular permeability was done as described by Sobeh et al. [7]. In brief, mice were treated orally with either *T. algeriensis* or *T. fontanesii* extracts in three dose levels (200, 400, and 600 mg/kg), diclofenac (20 mg/kg), dexamethasone (2 mg/kg) or vehicle. Evans blue (0.25% solution in normal saline, 0.2 mL) was injected in the tail vein 1 h later. Acetic acid (0.6% in normal saline, 1 mL/100 g) was then injected after 30 min in the peritoneal cavity of the treated mice. One group of mice was injected with normal saline to serve as normal control. Mice were euthanized 30 min later by cervical dislocation, the abdominal cavities were washed with 3 mL saline solution, and the collected washings were then centrifuged at 3000 rpm for 10 min. The vascular permeability is proportional to the absorbance of Evans blue dye in the supernatant, which was detected at 610 nm using a plate reader (Biotech, Winooski, VT, USA).

#### 2.4.4. Assay of Anti-Nociceptive Activity by Hot Plate Test

Evaluation of the possible central analgesic activity of the studied extracts was done by the hot plate test, according to Sobeh et al. [7]. Briefly, mice were divided into different groups (5 each) that received orally either the tested extracts in two dose levels (200 and 400 mg/kg), the vehicle (10 mL/kg), or the opioid analgesic nalbuphine (10 mg/kg) as a reference standard. One hour later, each individual mouse was placed on a hot plate pre-heated at 55 ± 1 °C and observed for any response to the heat-induced nociceptive pain (licking the fore and hind paws, hind paw lifting, or jumping). The latency time until the animal showed the first signs of discomfort was recorded, before (baseline) and at 1, 2, 3 and 4 h following the different treatments.

#### 2.4.5. Assay of Anti-Nociceptive Activity by Acetic Acid-Induced Abdominal Writhing

The peripheral analgesic activity of *T. algeriensis* and *T. fontanesii* extracts was evaluated by the acetic acid-induced writhing model in mice, according to Sobeh et al. [7]. Briefly, mice were divided into 4 groups (5–7 mice) that received orally, either the vehicle (1% Tween 80, 10 mL/kg), the extracts in two dose levels (200 and 400 mg/kg), diclofenac (20 mg/kg), or dexamethasone (2 mg/kg), 1 h prior to the intraperitoneal injection of 0.7% acetic acid (1 mL/100 g). The number of writhes, manifested as extension of hind legs, constriction of abdomen, or turning of trunk was recorded within 25 min.

#### 2.4.6. Anti-Pyretic Activity in Brewer’s Yeast Induced Pyrexia in Mice

Pyrexia in mice was induced as described by Liu et al. [8] with minor modifications. The initial rectum temperature of each animal was measured using a lubricated digital thermometer. Brewer’s yeast suspension was prepared in normal saline (30%) and injected subcutaneously behind the animal’s neck (1 mL/100 g). After 18 h, animals’ rectal temperatures were measured again (T_0_). Only animals that showed at least 0.5 °C rise in their rectal temperature were included in the study. Pyretic animals were treated orally with either *T. algeriensis* or *T. fontanesii* extracts in three dose levels (200, 400, and 600 mg/kg), paracetamol (150 mg/kg), or vehicle. Rectal temperatures were measured at 30 min, 1, 2, 3 and 24 h following the different treatments.

### 2.5. Molecular Modeling

Molecular modeling was performed through docking a sample of the extracts’ compounds to cyclooxygenase 1 (COX-1), cyclooxygenase 2 (COX-2), 5-lipoxygenase (5-LOX) and 5-lipoxygenase activating protein (FLAP). The X-ray crystallographic structures of the aforementioned enzymes (PDB codes: 5WBE, 5IKR, 3V99 and 2Q7M, respectively) were downloaded from the Protein Data Bank (www.rcsb.org). The docking protocol and visualization techniques were done using Molecular Operating Environment (2010.10; Chemical Computing Group Inc., Montreal, Canada). Downloaded proteins were cleared from all unnecessary repetitive amino acid chains or ligands, and proteins were protonated to ensure the availability of hydrogen atoms that were undetected during the crystallization processes. Docked compounds were either downloaded directly as sdf files from PubChem or drawn using MOE builder or saved as mol2 files, and all were compiled afterwards to one database. Compounds’ ionization was checked by washing them; bond lengths were scaled to reasonable values. Tautomeric states at pH 7.0 were checked and partial charges were adjusted. Energy minimization was done to all compounds using MMFF94x force field. In the docking protocol, structural water molecules within the vicinity of co-crystallized ligands were considered an integral part of the binding site, if available. Docking was done using the default settings of placement (Triangle Matcher) and scoring (London dG), and refinement was set to force field. The docked poses were evaluated based on various criteria; their root mean squared deviations from the co-crystallized ligand, their scores as an indication of their free energy of binding as well as their subsequent interactions with the surrounding amino acids.

### 2.6. Statistical Analysis

Analyzing data was done by the aid of GraphPad Prism software, version 6 (San Diego, CA, USA). Analysis of Variance (ANOVA) or repeated-measures analysis of variance (RM-ANOVA) followed by Tukey’s post hoc test and Student’s *t*-test were used to detect differences between groups. Data are presented as mean ± S.E.M.

## 3. Results and Discussion

**Figure 1:**
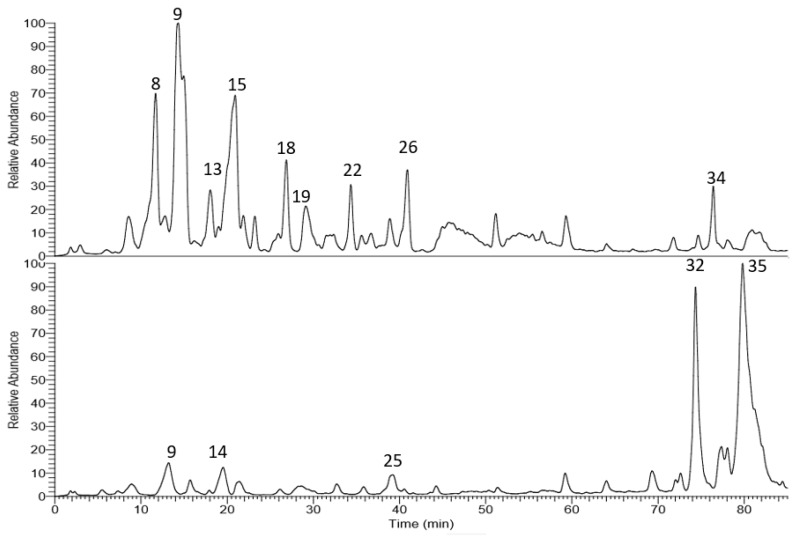
LC-MS base peak chromatogram of *T. algeriensis* (top) and *T. fontanesii* (bottom) leaves methanol extracts.

The corresponding LC-MS chromatograms of the methanol extracts from *T. algeriensis* and *T. fontanesii* leaves are presented in Figure 1. Chemical structures were elucidated based on HPLC-PDA, together with electrospray ionization mass spectrometry in the negative mode (ESI-MS and MS^n^). Compound identification was done based on their molecular ion peaks, MS^2^ fragmentation and comparison with data in literature. Table 1 summarizes the observed MS and MS/MS data and the relative abundance for each analyte within the studied extracts. Altogether, 35 secondary metabolites were tentatively identified in the extracts. Phenolic acids, mainly rosmarinic acid and its derivatives dominated *T. algeriensis* extract while the phenolic diterpene, carnosol, and the methylated flavonoid, salvigenin, was the major secondary metabolite in the *T. fontanesii* extract.

**Figure 2:**
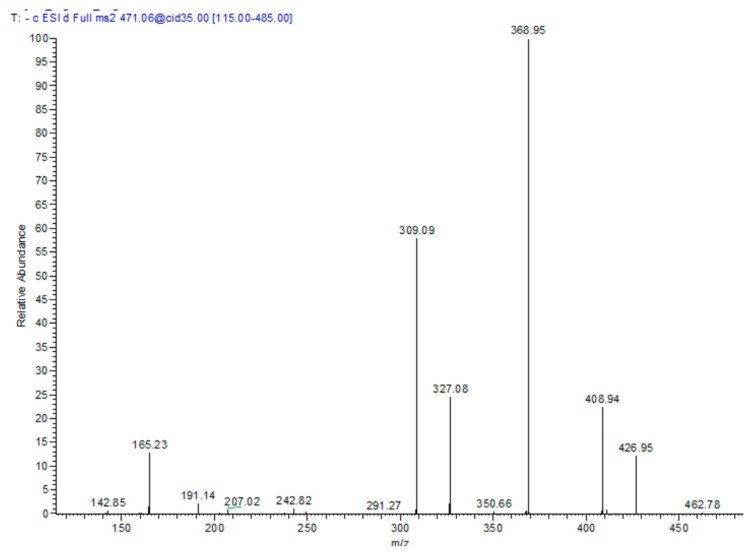
MS/MS spectra of compound 13: phloretic acid caffeoyl 3-hydroxy-3-methylglutaroyl in the negative ion mode.

In the *T. algeriensis* extract, the compound eluted at 17.76 min showed a molecular ion peak [M − H] *m*/*z* of 471 with daughter ions of 369 [M – H − 102], 327 [M − H − 144], and 309 [M − H − 162]. Losing the three fragments of 62, 102 and 144 amu marked the presence of 3-hydroxy-3-methylglutaryl, while the loss of 162 amu indicated the presence of caffeoyl moiety. The daughter ion at 165 suggested phloretic acid; therefore, the compound was assigned as phloretic acid caffeoyl 3-hydroxy-3-methylglutaroyl (Figure 2).

### 3.1. In Vitro Activities

#### 3.1.1. Inhibition of Cyclooxygenase (COX-1/2), Lipoxygenase (5-LOX), and Total Antioxidant Capacity (TAC) Assay

As shown in Table 2, *T. algeriensis* extract showed a higher selectivity for COX-2 than for COX-1 in vitro with a selectivity index similar to that of the selective COX-2 inhibitor, celecoxib (SI value = 248 and 266.2, respectively) and higher than both indomethacin and diclofenac (*p* < 0.05). Noteworthy, *T. fontanesii* extract was more selective for COX-2 (SI = 322) than both *T. algeriensis* and celecoxib, however this difference was not statistically significant (*p* > 0.05). Both *T. algeriensis* and *T. fontanesii* extracts exhibited similar potency like diclofenac to inhibit lipoxygenase in vitro and were also similar to the reference 5-LOX inhibitor; zileuton, Table 2.

Furthermore, *T. algeriensis* and *T. fontanesii* extracts revealed a high antioxidant potential: The total antioxidant capacity (TAC) of *T. fontanesii* was higher than that of *T. algeriensis* and ascorbic acid, the antioxidant reference standard, Table 2. The antioxidant and the anti-inflammatory effects of the extracts may be attributed to the reducing properties of the phenolic compounds which are dominant in the extracts. The major constituents of *T. algeriensis* are rosmarinic acid and its glucoside derivative (rosmarinic acid glucoside), luteolin glucuronide and salvianolic acid K. While the major constituents of *T. fontanesii* are carnosol and salvigenin. Both species contain flavonoids (quercetin, luteolin, apigenin and their derivatives) and phenolic acids (caffeic acid and phloretic acid and their derivatives).

#### 3.1.2. Effects of *T. algeriensis* and *T. fontanesii* Extracts on Carrageenan-Induced Paw Edema in Rats

**Figure 3:**
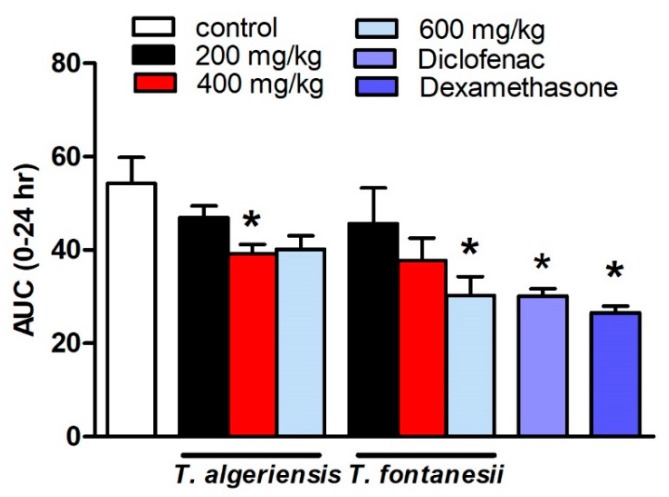
Effect of the pretreatment with *T. algeriensis* or *T. fontanesii* (p.o., 200, 400, and 600 mg/kg,), diclofenac (p.o., 20 mg/kg) or dexamethasone (p.o., 2 mg/kg) on carrageenan- (1% suspension, 0.1 mL)-induced hind paw edema in rats. Edema thickness (mm) was measured before and hourly for 5 h and at 24 h following carrageenan injection. Data represents the AUC_0-24_ and is presented as the mean ± S.E.M (*n* = 5). * *p* < 0.05 vs. control values by one-way ANOVA followed by Tukey post hoc test.

Several models to induce acute and chronic inflammation are widely reported. The most commonly used model of acute inflammation is carrageenan-induced paw edema that is commonly used for screening new anti-inflammatory drugs. The broad use of this model is attributed to the fact that it is sensitive to the cyclooxygenase inhibitors. The other reason is that this model was used to evaluate the effect of NSAIDs, which involves the inhibition of PGs synthesis. Several inflammatory mediators are released in subsequent phases to induce paw edema. In the initial or vascular phase, histamine, serotonin, and bradykinin are released [13]. They cause vasodilatation and extravasation. In the final or cellular phase, leukocytes migrate to the inflamed area [14]. Prostaglandins play a crucial role in the cellular phase, which takes place after about four hours following the intra-plantar administration of carrageenan [15]. The present study showed that the rats injected with carrageenan (0.1 mL, 1% in 0.9%, sub-planter) exhibited increased paw thickness, an indication of paw inflammation, when measured at hourly intervals for 5 h and at 24 h after injection. The later responses peaked at 3 h post-injection. Rats pretreated 1 h earlier with *T. algeriensis* or *T. fontanesii* extracts (200 and 400 mg/kg, p.o.) showed a mild reduction in edema thickness compared to the control rats. Increasing the dose of *T. algeriensis* extract to 600 mg/kg did not result in further inhibition of the measured edema thickness. On the other hand, *T. fontanesii* extract at the highest dose (600 mg/kg) produced a comparable effect to that obtained with the standard anti-inflammatory drug, diclofenac (20 mg/kg, p.o.) as both reduced paw thickness by 44% of the control AUC_0-24_ values. In addition, dexamethasone (2 mg/kg, p.o.) showed a 51% reduction in AUC_0-24_ values compared to the vehicle-treated rats (Figure 3). Carrageenan activates COX-2, which produces PGE2 using arachidonic acid as a precursor. PGE2 causes acute exudation, resulting in swelling [16]. The anti-inflammatory activities of both extracts are attributed to suppressing COX-2 and thus the inhibition of prostaglandin synthesis, which was confirmed by the in vitro and molecular docking studies. The higher potency of *T. fontanesii* extract relative to that of *T. algeriensis* could be explained by the higher selectivity of *T. fontanesii* extract towards COX-2 (SI = 322) than both *T. algeriensis* and celecoxib *in vitro*, however this difference was not statically significant (*p* > 0.05).

The anti-inflammatory effect of *T. algeriensis* may be related to its major active constituents, rosmarinic acid and rosmarinic glucoside. Previous studies showed that rosmarinic acid has potent anti-inflammatory effect through inhibition of NF-κB activation [17]. On the other hand, salvigenin is the major active constituent of *T. fontanesii.* It has a wide range of biological effects, including antioxidant, antitumor, antibacterial and vasorelaxant activities in isolated rat aortas, immunomodulatory activities, inhibition of the growth of malarial parasites, and potent anti-inflammatory effects [18,19].

#### 3.1.3. Effects on Carrageenan-Induced Leukocyte Migration into the Peritoneal Cavity in Mice

**Figure 4:**
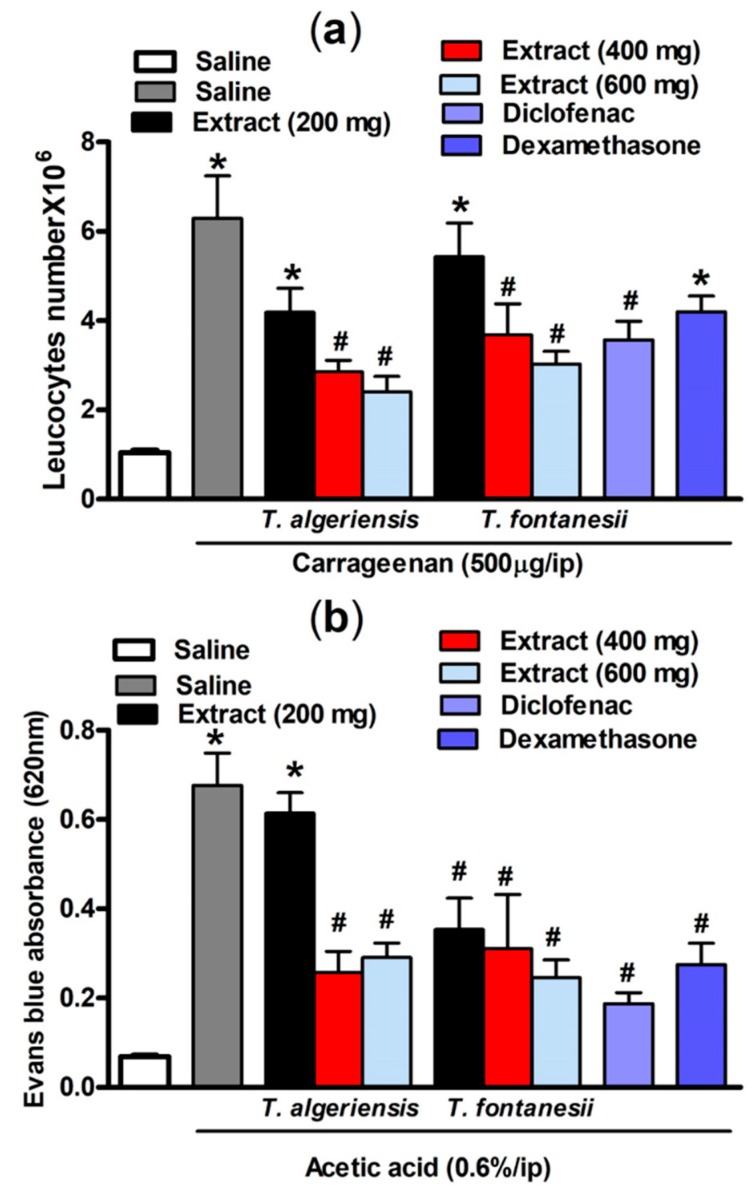
(**a**) Carrageenan (500 µg, i.p.) effect on leukocytes migration into the peritoneal cavity in mice (total number ×10^6^) with or without 1 h prior pretreatment with *T. algeriensis, T. fontanesii* (p.o., 200, 400, and 600 mg/kg), diclofenac (p.o., 20 mg/kg) or dexamethasone (p.o., 2 mg/kg). Data is presented as mean ± S.E.M (*n* = 5–7). * *p* < 0.05 vs. vehicle (saline) values, ^#^ *p* < 0.05 vs. control (carrageenan treated group). (**b**) Effect of *T. algeriensis* or *T. fontanesii* (p.o., 200, 400 and 600 mg/kg) on acetic acid-induced vascular permeability. The absorbance of Evans blue dye in the abdominal cavity exudate was measured to indicate the inflammation level. The values are presented as the mean ± SEM (*n* = 5–6). * *p* < 0.05 compared to saline group. ^#^ *p* < 0.05 compared to the saline group treated with acetic acid only by one-way ANOVA followed by Tukey post hoc test.

As shown in Figure 4, intraperitoneal injection of carrageenan- (500 μg/cavity, 0.1 mL)-induced leukocyte migration into the peritoneal cavity in mice. This can be shown as an increase in the total leukocyte number compared to the saline-treated mice (6.29 ± 0.95 vs 1.04 ± 0.06 leukocytes × 10^6^ mL^−1^). Mice pretreated with *T. algeriensis* or *T. fontanesii* extract (200, 400 and 600 mg/kg, p.o.) before the carrageenan challenge showed a dose-dependent reduction in the total leukocyte number that reached up to 62 and 52%, respectively compared to the vehicle when the highest dose (600 mg/kg) was used. Notably, the previous effect was stronger than that of diclofenac or dexamethasone, which showed 39 and 30% reductions in leucocyte number, respectively. It was reported previously that inhibition of leukocyte migration into the peritoneal cavity could be caused by either inhibiting the chemotactic substances’ expression and/or suppressing the adhesion molecule production [20]. In carrageenan-induced peritonitis, the inflammatory mediators such as prostaglandins and the proinflammatory cytokines are released into the peritoneal cavity by resident and endothelial cells in the peritoneum, such as macrophages and mast cells [21]. Suppression of the synthesis and release of these inflammatory mediators is apparently responsible for the protective effect of both extracts.

#### 3.1.4. Effects on Acetic Acid-Induced Vascular Permeability in Mice

Acetic acid-induced vascular permeability was conducted to assess the extracts’ activity against the first phase of inflammation. Acetic acid strongly enhances vascular permeability and facilitates Evans blue dye vascular leakage. Our experimental results demonstrated that acetic acid injection (0.6%, i.p.) in mice resulted in an increased vascular permeability demonstrated by the significantly (*p* < 0.001) higher Evans blue absorbance compared to the saline-injected mice (0.68 ± 0.07 vs 0.07 ± 0.004). Acetic acid causes an increase in the level of prostaglandins, histamine, and serotonin in peritoneal fluids, which in turn leads to vasodilation and increased vascular permeability [22]. This effect was attenuated in mice pretreated 1 h prior acetic acid injection with *T. algeriensis* extract (400 or 600 mg/kg, p.o.) by 63 and 58%, respectively. On the other hand, the *T. fontanesii* extract was protective at all dose levels (200, 400 and 600 mg/kg, p.o.) by 50, 55 and 64%; and produced a greater reduction in Evans blue reading compared to *T. algeriensis.* This is matched with the in vitro results that showed a stronger effect for *T. fontanesii* extract than *T. algeriensis* extract on COX-2. Based on this outcome, the anti-inflammatory effect of both extracts on the acute inflammation phase might be attributed to the inhibition of the release of inflammatory mediators and inhibition of vasodilation. Notably, exudates from diclofenac (20 mg/kg) or dexamethasone (2 mg/kg) treated mice showed 73 and 60% lower Evans blue readings, respectively compared to the control (Figure 4).

#### 3.1.5. Effects on Acetic Acid-Induced Writhing and Hot Plate Test in Mice

**Figure 5:**
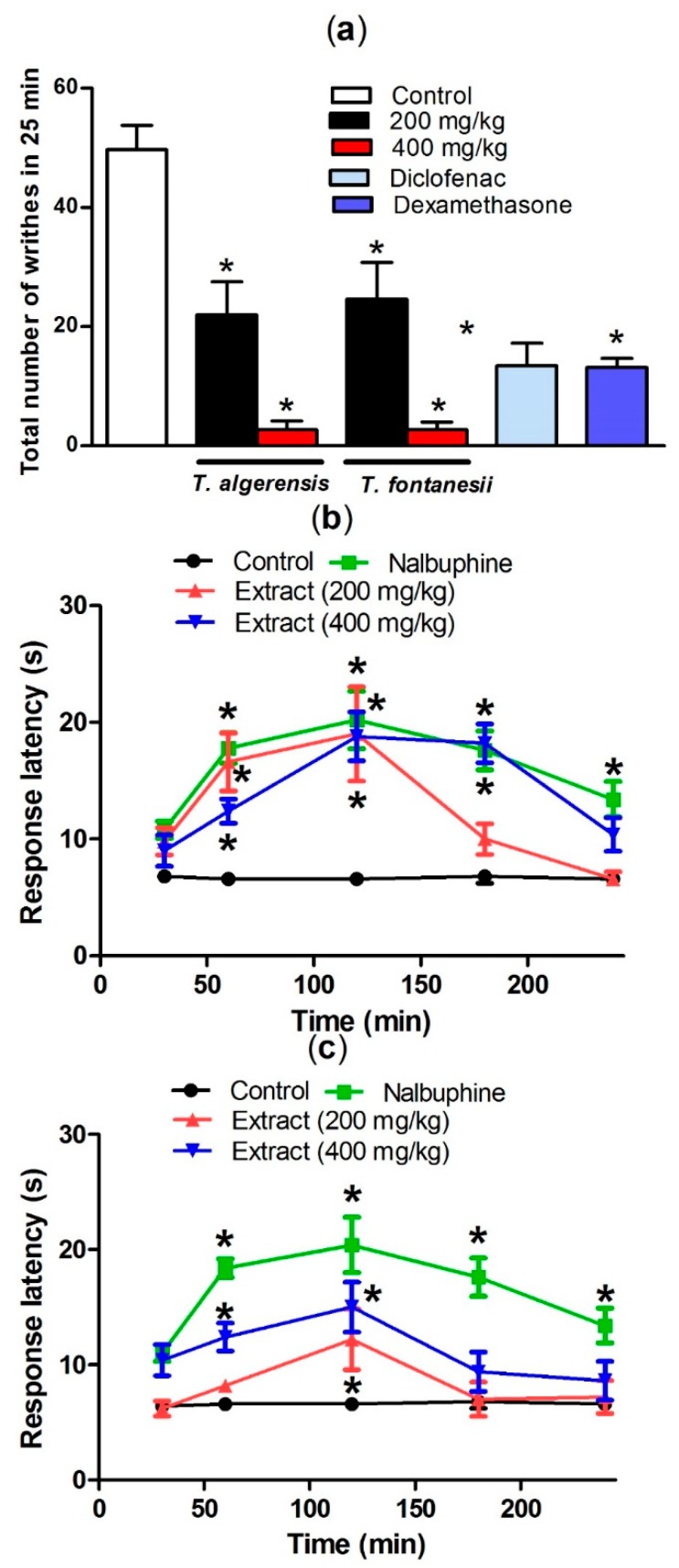
Effect of *T. algeriensis* or *T. fontanesii (p.o.,* 200 and 400 mg/kg), diclofenac (p.o., 20 mg/kg) or dexamethasone- (p.o., 2 mg/kg) on acetic acid (0.7%, 1 mL/100 g)-induced writhing in mice (**a**). Effect of *T. algeriensis* on hot plate response latency (**b**). Effect of *T. fontanesii* on hot plate response latency (**c**). Response latency in (s) was measured 1–4 h after the different extracts (200 and 400 mg/kg, p.o.), vehicle or nalbuphine (p.o., 10 mg/kg). Data is presented as mean ± S.E.M (*n* = 5–8). * *p* < 0.05 vs. control values by one-way ANOVA followed by Tukey post hoc test.

Acetic acid injection induced painful response in mice represented by the abdominal writhing response. The production of prostaglandins, specifically PGF2α and PGE2, in the peritoneal fluids from arachidonic acid that is mediated by cyclooxygenase enzymes, upon injecting acetic acid, stimulates the local peritoneal receptors leading to the abdominal writhing response. Leukotrienes are produced as well via the lipoxygenase enzyme from arachidonic acid [23]. Both prostaglandins and lipoxygenase products induce pain and inflammation and increase capillary penetrability. As shown in Figure 5, oral pretreatment of mice with *T. algeriensis* or *T. fontanesii* extracts in the two dose levels 1 h prior to acetic acid injection showed a similar significant peripheral analgesic activity represented as a dose-dependent decrease in acetic acid-induced writhes in mice. The effect achieved by the higher dose (400 mg/kg) of both extracts was superior to that observed by diclofenac (20 mg/kg), or dexamethasone (2 mg/kg) as almost 94% reduction of the writhing response was observed (Figure 5).

The analgesic effect of extracts in the present study may be mediated via inhibition of synthesis of prostaglandin and leukotrienes synthesis. These results strongly endorse the fact that both extracts exhibit peripheral analgesic effects, presumably, through locally inhibiting the peritoneal receptors, which contribute then to the inhibition of cyclooxygenase. Previous studies showed that salvigenin, the major active constituent of *T. fontanesii,* has a significant analgesic effect like morphine [18].

Hot plate test was done to evaluate the central anti-nociceptive activity. Both extracts showed a significant central analgesic effect suggesting the presence of centrally active anti-nociceptive components. The animals pretreated with any of the extracts (200 and 400 mg/kg, i.p.), showed a longer response latency in a dose-dependent manner. Surprisingly, *T. algeriensis* extract has greater central analgesic activity compared to *T. fontanesii* at both tried doses. Additionally, the higher dose (400 mg/kg) of *T. algeriensis* showed a similar effect to nalbuphine, the narcotic analgesic with central activity that was used as a reference standard in this experiment (Figure 5). It is well reported that flavonoids have a potential to cross the blood–brain barrier and affects the opioid and other receptor types in the CNS [18]. These data indicated that the opioid system could be involved in the antinociception exerted by both extracts.

#### 3.1.6. Effect of Extracts on Brewer’s Yeast Induced Pyrexia in Mice

Fever represents a brain mediated response to injury or infections by elevating body temperature. This response is elicited by exogenous pyrogens and promoted by the endogenous ones such as IL-1β, IL-6, TNF-α, endothelin-1 (ET-1), corticotrophin release factor (CRF), bradykinin, preformed pyrogenic factor (PFPF), and prostaglandin E2 (PGE2) [24]. PGE2-dependent fever results mainly by the action of the activated cyclooxygenase enzymes in the preoptic zone of the hypothalamus [25]. Brewer’s yeast injection in mice raised their rectal body temperature when measured 18 h following the injection (Table 3). Mice treated with *T. algeriensis* (200, 400 and 600 mg/kg, p.o.) did not show any significant antipyretic effect compared to the vehicle-treated mice, although there is a trend to lower body temperatures at the highest used dose (600 mg/kg). On the other hand, mice treated with *T. fontanesii* (200 and 400 mg/kg, p.o.) showed mild antipyretic effect that started 24 h and 3 h post-treatment, respectively. The 600 mg/kg dose of the extract showed a much stronger antipyretic profile with a faster and prolonged effect that started 1 h post treatment and extended up to 24 h (Table 3). Carnosol, a major constituent of *T. fontanesii*, was reported to increase the gene expression of heat shock proteins namely, hsp-16.1 and hsp-16.2 and the antioxidant enzyme, SOD in *Caenorhabditis elegans* [26]. The increase in heat shock protein expression is beneficial to improve stress resistance under heat stress and other stresses [27]. Carnosol is the product of the oxidative degradation of carnosic acid [28]. The bioavailability and metabolism of carnosol is similar to carnosic acid [29]. Previous studies showed that it is well absorbed after oral administration and reachs the blood stream and is available for several hours after administration. This could explain why the antipyretic effect extended to 24 h post-treatment. In addition, carnosol inhibits protein kinase C pathway and blocks the binding of AP-1 to the COX-2 promoter, thus inhibits prostaglandin synthesis [30]. The ability of the studied extracts to reduce the mice’s body temperature indicates well regulation of the PGE2-dependent fever in the hypothalamus, an effect that was confirmed by the in vitro study.

### 3.2. Molecular Docking

Out of the compounds identified in our extracts, twenty-two compounds were selected as candidates for the docking study to estimate the anti-inflammatory potential of the studied extracts. The compounds selected for the docking process are listed in Table 4**,** along with their docking scores on the inflammatory proteins of concern. Compounds were selected based on either the abundance of the compound in the extract, or the structural features of the compound, particularly the presence of an acidic functional group as a reported key structural element in various anti-inflammatory lead compounds.

#### 3.2.1. Cycloxygenase-1

Cycloxygenase active site is mainly created by a long hydrophobic channel, with Ser530 and Tyr385 at its apex [31]. Other important amino acids are Arg120, Tyr355, and Glu524, all of which form the constriction at the bottom part of the channel [32]. This constriction opens up paving the way for substrates and inhibitors entry to the channel. The catalytic activity of COX-1 is mainly mediated by trapping arachidonic acid (AA) between residues Arg120, Tyr355 and the catalytic Tyr385 [33].

Docking of the 22 compounds into the active site of COX-1 was performed (PDB code: 5WBE; complex of mofezolac-COX1). The co-crystallized ligand mofezolac, as well as diclofenac, were simultaneously docked for the purpose of validating our docking protocol. Mofezolac is reported to participate in a salt bridge interaction with the guanidium group of Arg120 located at the entrance of the hydrophobic active site channel, similar to arachidonic acid carboxylate which ion pairs with the same residue [34]. Furthermore, mofezolac is found to participate in hydrogen bonding to Tyr355. It is also surrounded by a rich set of hydrophobic residues in COX1 binding site, which explain its numerous van der Waal interactions [33].

**Figure 6:**
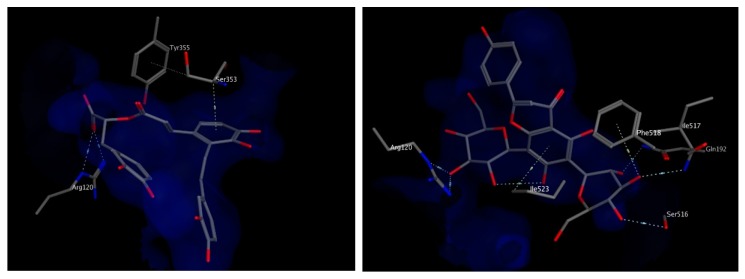
3D view of interactions of salvianolic acid A (**left**) and apigenin 6,8-di-C-hexosides (**right**) with amino acid residues of COX-1. Side chains of amino acids were omitted for clarity, except for Ser353 (left) and Ile517 (right), whose backbones participate in hydrophobic interactions with the docked compounds.

The docking study was verified by examining the amino acids interacting with mofezolac and diclofenac, which were found to be Arg120, Tyr 355, Phe518 and Ile523. As for the molecules identified from the two plants extracts, the top scoring compounds were salvianolic acid A and apigenin 6,8 di-*C*-hexoside, the latter is relatively more abundant in the extract of *T. algeriensis*. The chosen docked poses for the two compounds scored −21.08 kcal/mol and −20.48 kcal/mol, respectively, relative to −13.08 kcal/mol for diclofenac and −14.60 kcal/mol for mofezolac. Salvianolic acid A binds to COX-1 through hydrogen bonding to Arg120 and hydrophobic interactions with Ser353. On the other hand, the binding of apigenin 6,8-di-*C*-hexosides inside COX-1 was fortified by the interactions with several surrounding amino acids including hydrogen bonds with Arg120, Ser 516, Ile 517 and Gln192 in addition to the hydrophobic interactions with Phe518 as well as Ile523 (Figure 6). Most of the interactions were found to be established by the two glucoside moieties.

#### 3.2.2. Cycloxygenase-2 Protein

The crystal structure of mefenamic acid complexed with COX-2 enzyme (PDB ID: 5IKR) was utilized in the current docking process. The downloaded protein consists of 2 identical polypeptide chains, with 551 amino acid residues each [31]. It has been revealed in the structure of the non-productive complex of arachidonic acid with COX-2, that the substrate adopts an inverted conformation inside the binding site, where the carboxylate moiety interacts with Ser530 and Tyr385. Other inhibitors such as diclofenac and lumiracoxib, have been reported to bind in the same inverted conformation to Ser530 and Tyr385, highlighting the importance of those 2 residues in ligand association [32]. Contrary to COX-1, Arg120 mutation to an uncharged residue doesn’t lead to detrimental loss of complex stability of COX-2 inhibitors, suggesting that interaction of arachidonic acid with Arg120 does not play a great role in COX-2 as it does in the case of COX-1 [33].

**Figure 7:**
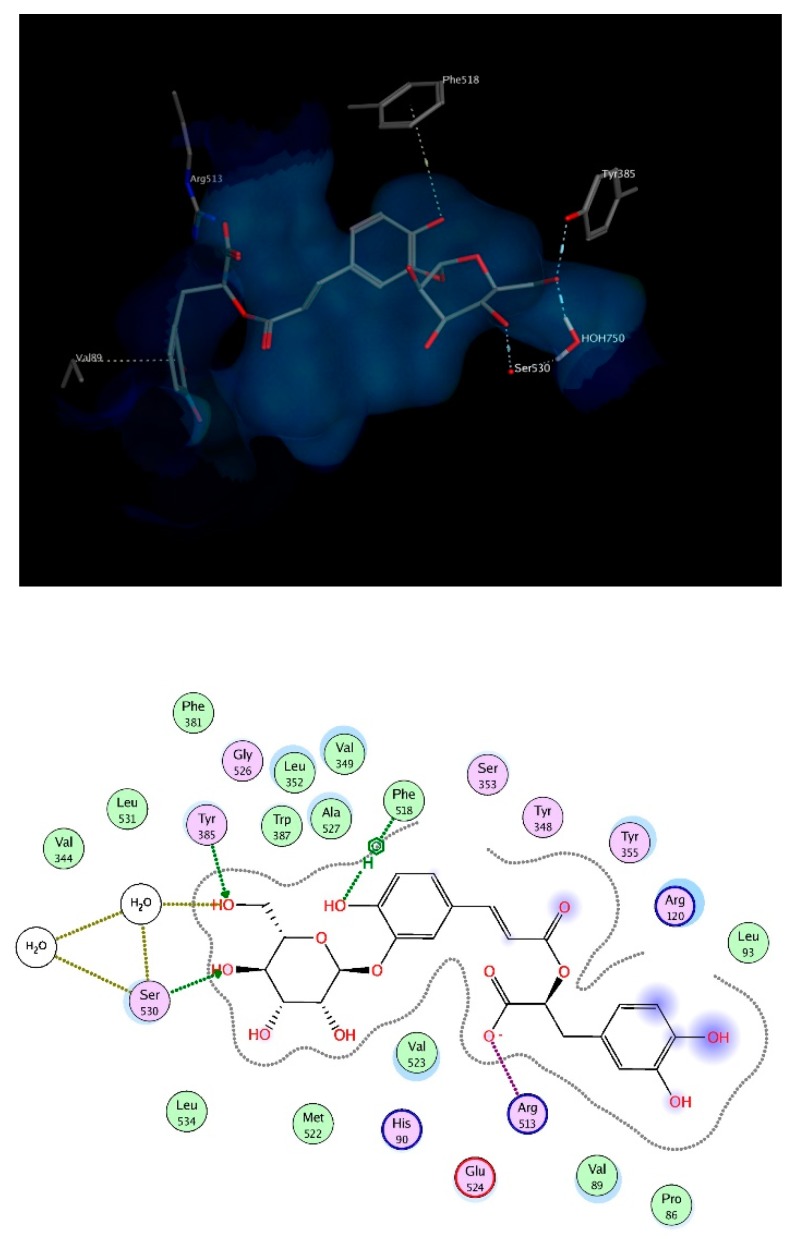
3D (top) and 2D (bottom) interactions of rosmarinic acid glucoside with amino acid residues of COX-2. In the 3D view, side chains of amino acids were omitted for clarity.

Mefenamic acid binds to COX-2 enzyme through hydrogen bonding to Ser530 and Ty355. Re-docking mefenamic acid as well as the known COX-2 inhibitor, celecoxib retrieved binding modes that reproduced the interactions with the exact 2 amino acids. Docking the chosen compounds found in *T. algeriensis* and *T. fontanesii* revealed that the top scoring compound was rosmarinic acid glucoside. The free binding energy for the best docking pose scored −25.9 kcal/mol, relative to −11.53 kcal/mol and −15.28 kcal/mol for mefenamic acid and celecoxib, respectively. The complex of rosmarinic acid glucoside and COX-2 was stabilized by hydrogen bond interactions to Ser530 (directly and indirectly via structural water molecules) as well as Tyr385, it was further strengthened by ionic interaction of the carboxylate oxygen with Arg513 as well as hydrophobic interactions with Phe518 and Val89, (Figure 7). In view of the docking scores summarized in Table 4, it is obvious that COX-2 is plausibly the most important target for the docked compounds. This can be deduced from the better scores obtained for the compounds on COX-2 relative to the other involved enzymes. It is worth noting that the molecular docking results were supported by the in vitro results.

#### 3.2.3. -Lipoxygenase Protein

The only X-ray crystal structure which represents a substrate-bound form of 5-LOX was chosen for docking (PDB ID:3V99). In this complex, 5-LOX is bound to its natural substrate, arachidonic acid. The catalytic center of 5-LOX mainly consists of hydrophobic amino acids, such as Ile406, Ala410, Leu414, Ile425, Val604 and Leu607. The carboxylic group of arachidonic acid seems to interact with Phe177 of 5-LOX, in addition to the hydrophobic contacts [35].

**Figure 8:**
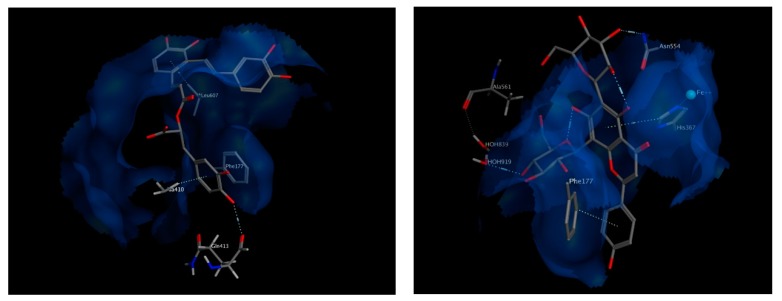
3D view of interactions of Salvianolic acid A as well as Apigenin 6,8-di-C-hexosides with amino acid residues of 5-LOX. Side chains of amino acids were omitted for clarity, except for Gln413 and Ala561, whose backbones participate in hydrogen bonding with the docked compounds.

The best chosen two compounds were salvianolic acid A and apigenin 6,8 di-*C*-hexosides, with binding free energy values of −20.87 and −22.19 kcal/mol, respectively. Those two compounds scored even better than the re-docked arachidonic acid (−11.6 kcal/mol) and the known inhibitor, Zileuton (−11.04 kcal/mol). Salvianolic acid A interacts via hydrogen bonding to Gln413 and via pi interactions with Phe177 and Leu607. It also interacts hydrophobically with Ala410. On the other hand, apigenin 6,8 di-*C*-hexoside interacts via pi-interactions to Phe177 as well as His367 which is in turn coordinated to the iron atom available in the middle of the active site. It also interacts by a hydrogen bond to Asn554, and indirectly to Ala561 through nearby water molecules. The interactions of both compounds within the active site of 5-LOX suggest their strong potential as high affinity inhibitors (Figure 8).

#### 3.2.4. 5 Lipoxygenase Activating Protein (FLAP)

FLAP has a critical role in biosynthesis of leukotrienes. It delivers arachidonic acid to 5-lipoxygenase for subsequent conversion to leukotriene A4 followed by the generation of other pro-inflammatory leukotrienes. Reported crystal structures of FLAP reveal that it forms homotrimers, where each monomer contains four transmembrane α-helices. The binding site is embedded within the membrane, formed by the interface of α -helices 2 and 4 of one monomer and α -helix 1 of the neighboring monomer. Each ligand molecule is bound to the groove at the interface between two adjacent monomers [36,37].

Important amino acids forming hydrophobic interactions with the bound ligand include residues Val20, Val21, Gly24, Phe25, Ala27, Tyr112, Ile113, A63, Ile119, Leu120 and Phe123. Few polar interactions are also observed with residues Asn23 from transmembrane helix α1, residues Asp62 and Thr66 from transmembrane helix α2 and residue Lys116 from transmembrane helix α4 of the neighboring FLAP monomer [36].

**Figure 9:**
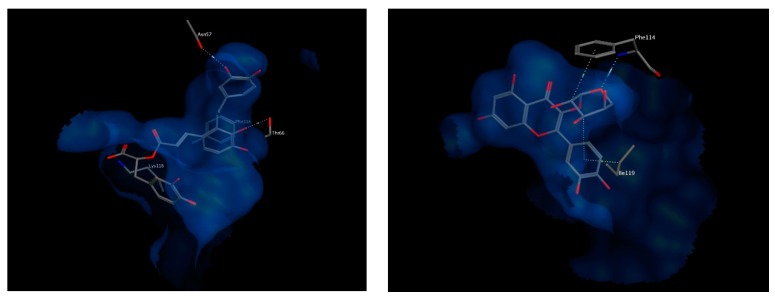
3D view of interactions of Salvianolic acid A (left) as well as Quercetin pentoside (right) with amino acid residues of FLAP. Side chains of amino acids were omitted for clarity, except for Thr66 (right) and Phe114 (left), whose backbones participate in hydrogen bonding with the docked compounds.

The best chosen two compounds were salvianolic acid A and quercetin pentoside with binding free energy values of −17.37 and −16.79 kcal/mol, respectively. Re-docking the bound inhibitor MK-591 resulted in a score of −11.57 kcal/mol. Salvianolic acid A undergoes polar interactions with residues Lys116, Thr66, and Asn57, while quercetin pentoside interacts with Phe114 via hydrogen bonding with the glucosidic oxygen, in addition to hydrophobic interactions with Phe114 and Ile119. Interactions of both compounds are shown in Figure 9. Because the docked compounds are expected to be partially ionized in the physiological pH, the docking experimented were made for the ionizable forms which showed some ionic interactions with the basic amino acids in the binding sites of the target enzymes such as lysine and arginine.

## 4. Conclusions

The current investigation showed that the major constituents of *T. algeriensis* are rosmarinic acid and its glucoside derivative (rosmarinic acid glucoside), luteolin glucuronide and salvianolic acid K, while the major constituents of *T. fontanesii* are carnosol and salvigenin. Both species contain flavonoids (quercetin, luteolin, apigenin, and their derivatives) and phenolic acids (caffeic acid and phloretic acid and their derivatives). Both studied *Thymus* species exhibited antioxidant, anti-inflammatory, analgesic, and antipyretic effects in the performed in silico, in vitro, and in vivo experiments, which may be attributed to their phenolic compounds. They are a more potent antioxidant than ascorbic acid and more selective against COX-2. *T. fontanesii* tends to be a more potent COX-2 inhibitor than *T. algeriensis* and similar to celecoxib, the selective COX-2 inhibitor, indicating more potent anti-inflammatory, analgesic, and antipyretic effects. Molecular docking data showed that the docked compounds gave better scores on COX-2 than COX-1 and other enzymes involved in the study, which was supported by the in vitro results obtained. The docking poses retrieved in the binding sites of inflammatory proteins of concern were similar to co-crystallized ligands’ orientations and were able to reproduce the same interactions with surrounding amino acids. Top candidates from our in silico study included salvianolic acid A, rosmarininc acid glucoside, apigenin 6,8-di-C-hexosides as well as quercetin pentoside. These candidates represent leads of natural origin for developing safer anti-inflammatory alternatives to the currently in-use drugs. In conclusions, *Thymus algeriensis* and *Thymus fontanesii* are good candidates to counteract inflammation and reactive oxygen species-related diseases. Their pharmacologocical properties are still in need for further in vivo examinations.

## Figures and Tables

**Table 1:** Secondary metabolites from *T. algeriensis* and *T. fontanesii* leaves extracts.

No.	Rt	M-H	MS/MS	Relative Abundance		Proposed Compounds
				*T. algeriensis*	*T. fontanesii*	
1	1.46	191		+	-	Quinic acid ^a^
2	1.60	133	115	+	-	Malic acid
3	1.87	341	179	+	+	Caffeic acid glucoside ^a,b^
4	2.66	305	97, 225	+	+	Gallocatechin ^a^
5	8.58	387	163, 179, 369	+	+	12-Hydroxyjasmonic acid 12-O-hexoside
6	9.47	165	99, 149, 165	+	+	Phloretic acid
7	10.55	493	161, 179, 359	+	+	Salvianolic Acid A
8	11.68	555	161, 359, 493	+++	+	Salvianolic acid K ^c^
9	12.85	521	161, 359	+++	+	Rosmarinic acid glucoside ^c^
10	14.37	359	161, 179, 197	+	+	Rosmarinic acid ^a,c^
11	16.90	385	153, 161, 223	-	+	Sinapic acid glucoside
12	17.58	623	179,315, 447	+	-	Isorhamnetin pentosyl-glucuronide
13	17.76	471	165, 309, 369	+	-	Phloretic acid caffeoyl 3-hydroxy-3-methylglutaroyl
14	18.17	327	161, 165	-	+	Caffeoyl-phloretic acid
15	20.12	461	175, 285	+++	+	Luteolin glucuronide ^c^
16	21.55	593	269, 353, 413, 473	+	+	Apigenin 6,8-di-*C*-hexosides ^c^
17	25.79	449	151, 287	+	-	Eriodictyol glucoside ^c^
18	26.91	551	161, 359, 519	++	+	Schizotenuin F
19	29.13	549	161, 387, 531	+	+	Caffeoyl ethylrosmarinate
20	32.33	303	125, 177, 285	+	+	Dihydroquercetin
21	34.26	563	193, 387, 531	+	+	Feruloyl ethylrosmarinate
22	35.53	637	285, 461	++	-	Luteolin feruloyl glucuronide
23	35.78	609	301, 447	-	+	Querectin rhamnosyl-glucoside ^c^
24	38.52	579	285, 417, 447	+	-	Luteolin pentosyl-glucoside
25	38.85	433	301	-	+	Quercetin pentoside
26	40.79	447	161, 285	++	+	Luteolin glucoside
27	43.97	329	125, 229, 329	-	+	Thymusin
28	44.75	417	161, 285, 417	+	-	Luteolin pentoside
29	51.34	287	151, 269	+	+	Eriodictyol ^d^
30	59.52	271	151, 177	+	+	Naringenin ^a,e^
31	64.01	269	149, 225, 269	-	+	Apigenin ^a^
32	74.31	329	270, 286, 329	+	++++	Carnosol ^a,b^
33	76.29	283	179, 266, 283	+	+	Genkwanin
34	77.36	343	179, 300, 325	+	+	Xanthomicrol
35	79.16	327	285, 299, 327	-	++++	Salvigenin

^a^ Previously identified from *T. fontanesii* [9]. Previously identified from *T. algeriensis*: ^b^ [10], ^c^ [3], ^d^ [11], ^e^ [12]. (-) = absent, (+) = minor, (++) = moderate and (+++ or ++++) = major based on the assigned extract.

**Table 2:** In vitro cyclooxygenase (COX-1 and COX-2) and lipoxygenase (5-LOX) inhibition (IC_50_) and the total antioxidant capacity (U/L) of *T. algeriensis* and *T. fontanesii* extracts.

Treatment	COX-1	COX-2	SI	5-LOX	TAC
	IC_50_ (µM)			IC_50_ (µM)	U/L
*T. algeriensis* Extract	12.4 ± 0.49	0.05 ± 0.01 *	248	2.70 ± 0.23	39.27± 3.47
*T. fontanesii* Extract	12.88 ± 0.94	0.04 ± 0.002 *	322	2.5 ± 0.4	44.33 ± 4.6 ^@^
Celecoxib	15.97 ± 1.03	0.06 ± 0.01 *	266.2	-	-
Diclofenac	4.06 ± 0.22	0.76 ± 0.06	5.34	2.60 ± 0.21	-
Indomethacin	0.1 ± 0.01	0.72 ± 0.06	0.14	-	-
Zileuton	-	-	-	3.20 ± 0.15	-
Ascorbic Acid	-	-	-	-	27.6 ± 1.40

Values are mean ± SEM. SI is COX selectivity index calculated as IC_50_ (COX-1)/IC_50_ (COX-2). * Significantly different from diclofenac and indomethacin, ^@^ significantly different from ascorbic acid at *p* < 0.05 by One-way analysis of variance followed by Tukey *post hoc* test, n = 3.

**Table 3:** Effect of *T. algeriensis* and *T. fontanesii* extracts on Brewer’s yeast induced pyrexia in mice.

Extract	Dose (mg/kg)	Rectal Temperature ^#^	Rectal Temperature Recorded Following Different Treatments				
			30 min	1 h	2 h	3 h	24 h
Control	-	38.36 ±0.27	38.68 ± 0.15	38.66 ± 0.19	38.80 ± 0.20	38.84 ± 0.30	38.26 ± 0.18
*T. algeriensis*	200	38.78 ± 0.12	38.56 ± 0.31	38.80 ± 0.40	39.00 ± 0.23	38.46 ± 0.20	37.88 ± 0.53
	400	38.06 ± 0.52	38.14 ± 0.34	38.54 ± 0.29	38.54 ± 0.19	38.24 ± 0.16	37.52 ± 0.26
	600	39.18 ± 0.34	38.58 ± 0.29	37.85 ± 0.30	37.8 ± 0.25	38.10 ± 0.15	37.73 ± 0.18
*T. fontanesii*	200	38.42 ± 0.29	37.90 ± 0.48	38.44 ± 0.42	38.52 ± 0.15	38.30 ± 0.13	37.28 ± 0.16 *
	400	38.52 ± 0.37	38.14 ± 0.28	38.44 ± 0.37	38.02 ± 0.09	37.82 ± 0.11 *	37.24 ± 0.22 *
	600	38.48 ± 0.24	38.15 ± 0.14	37.58 ± 0.15 *	37.35 ± 0.16 *	37.6 ± 0.12 *	37.10 ± 0.23 *
Paracetamol	150	38.66 ± 0.18	38.18 ± 0.20	37.56 ± 0.30	37.06 ± 0.29 *	36.94 ± 0.25 *	36.54 ± 0.23 *

Values are presented as the mean ± S.E.M (n = 5), * *p* < 0.01 vs. control values. By one-way ANOVA followed by Tukey post hoc test. ^#^ Measured at 18 h after yeast injection.

**Table 4:** Docking scores retained from positioning selected compounds of *T. algeriensis* and *T. fontanesii* extracts into the binding sites of COX-1, COX-2, 5-LOX, and 5-Lipoxygenase Activating Protein (FLAP).

Compound Name	Docking Score (kcal/mol)			
	COX-1	COX-2	5-LOX	FLAP
Quinic acid	−9.29	−11.98	−13.78	−9.20
Malic acid	−9.54	−9.89	−8.85	−7.48
Caffeic acid glucoside	−15.79	−18.14	−12.90	−16.35
Phloretic acid	−9.39	−10.55	−9.40	−12.37
Salvianolic Acid A	−21.08	−23.32	−20.87	−17.37
Salvianolic acid K	−19.21	−24.15	−19.25	−13.77
Rosmarinic acid glucoside	−17.00	−25.93	−10.60	−14.82
Rosmarinic acid	−16.20	−19.46	−12.72	−15.09
Sinapic acid glucoside	−16.87	−20.64	−18.27	−16.00
Phloretic acid caffeoyl 3-hydroxy-3-methylglutaroyl	−16.88	−22.27	−1.06	−10.56
Caffeoyl-phloretic acid	−16.63	−18.03	−19.66	−14.35
Apigenin 6,8-di-*C*-hexosides	−20.48	−25.36	−22.19	−14.06
Schizotenuin F	−15.91	−25.75	−19.45	−14.92
Caffeoylethylrosmarinate	−18.63	−23.83	−19.27	−15.37
Feruloyl ethylrosmarinate	−15.63	−22.04	−14.17	−14.62
Quercetin pentoside	−17.20	−18.85	−19.57	−16.79
Luteolin glucoside	−17.89	−24.48	−18.46	−14.39
Naringenin	−13.96	−14.61	−11.34	−12.80
Carnosol	−12.78	−13.47	−12.88	−13.23
Genkwanin	−13.68	−14.47	−10.61	−11.22
Xanthomicrol	−15.16	−16.07	−14.87	−14.71
Salvigenin	−17.62	−17.23	−14.15	−12.42
